# Effects of porcine *MyD88* knockdown on the expression of TLR4 pathway-related genes and proinflammatory cytokines

**DOI:** 10.1042/BSR20160170

**Published:** 2016-11-17

**Authors:** Chaohui Dai, Li Sun, Lihuai Yu, Guoqiang Zhu, Shenglong Wu, Wenbin Bao

**Affiliations:** * Key Laboratory for Animal Genetics, Breeding, Reproduction and Molecular Design of Jiangsu Province, College of Animal Science and Technology, Yangzhou University, Yangzhou, Jiangsu 225009, China; † College of Veterinary Medicine, Yangzhou University, Yangzhou, Jiangsu 225009, China

**Keywords:** gene silencing, myeloid differentiation protein 88 (*MyD88*), pig, proinflammatory cytokines, Toll-like receptor (TLR)/Interleukin (IL)-1R pathway

## Abstract

As a critical adapter protein in Toll-like receptor (TLR)/Interleukin (IL)-1R signalling pathway, myeloid differentiation protein 88 (MyD88) plays an important role in immune responses and host defence against pathogens. The present study was designed to provide a foundation and an important reagent for the mechanistic study of *MyD88* and its role TLR/IL-1R signalling pathways in porcine immunity. Lentivirus-mediated RNAi was used to generate a porcine PK15 cell line with a silenced *MyD88* gene and quantitative real-time PCR (qPCR) and Western blotting were used to detect changes in the expression of critical genes in the Toll-like receptor 4 (TLR4) signalling pathway. ELISA was used to measure the levels of seven proinflammatory cytokines–interleukin-1β (IL-1β), tumour necrosis factor-α (TNF-α), IL-6, IL-8, IL-12, macrophage inflammatory protein (MIP)-1α and MIP-1β–in cell culture supernatants after *MyD88* silencing. We successfully obtained a PK15 cell line with 61% *MyD88* mRNA transcript down-regulated. In PK15 cells with *MyD88* silencing, the transcript levels of *TLR4* and *IL-1β* were significantly reduced, whereas there were no significant changes in the expression levels of cluster of differentiation antigen 14 (*CD14*), interferon-α (*IFN-α*) or *TNF-α*. The ELISA results showed that the levels of most cytokines were not significantly changed apart from IL-8 without stimulation, which was significantly up-regulated. When cells were induced by lipopolysaccharide (LPS) (0.1 μg/ml) for 6 h, the global level of seven proinflammatory cytokines up-regulated and the level of IL-1β, TNF-α, IL-6, IL-8 and IL-12 of Blank and negative control (NC) group up-regulated more significantly than RNAi group (*P*<0.05), which revealed that the *MyD88* silencing could reduce the TLR4 signal transduction which inhibited the release of proinflammatory cytokines and finally leaded to immunosuppression.

## INTRODUCTION

Toll-like receptors (TLRs) were found from *Drosophila* Toll proteins, in addition to participating in regulating the polarity formation of embryo flies back ventral, the receptors directly mediate flies’ natural (inherent) immune response to microbial infection. So far, there are 13 members in the TLRs family identified in mammals, which formed a huge type I transmembrane receptor protein family. Research showed that TLRs played an important role in defence mechanism of activating innate immunity and inducting adaptive immune, especially in innate immunity congenital immunity [1,2]. Toll-like receptor 4 (TLR4) signalling pathway is the essential member in TLRs family, which plays an important role in a variety of inflammatory reaction especially in diarrhoea and hydropsy of weaned piglets infected by pathogenic *Escherichia coli* stimulated by LPS [3,4]. And the TLR4 signalling pathway transmits signals through both myeloid differentiation protein 88 (MyD88)-dependent and -independent pathways. Therefore, the functional analysis of the *MyD88* gene is conductive to characterize the molecular mechanism that leads to diarrhoea and hydropsy of weaned piglets infected by pathogenic *E. coli*.

MyD88 is an important adapter protein in the TLR/Interleukin (IL)-1 receptor signalling pathway. It is a cytoplasmic soluble protein that contains an N-terminal death domain (DD) and a C-terminal Toll/Interleukin-1 receptor (TIR) domain [5]. The TIR domain plays a critical role in MyD88 signal transduction. MyD88 sends signals to downstream via the interaction between its TIR domain and TIR domain of membrane receptors. And performance for activation signals is similar between different receptors regulated by MyD88. The TIR domain of MyD88 can combine with the TIR domain of TLR or IL-1R to activate Interleukin receptor associated kinase (IRAK)-1/4 and TNF receptor associated factor-6 (TRAF-6). IRAK-1/4 and TRAF-6 can be activated when the TIR domain of MyD88 binds to the TIR domain of TLRs or IL-1R, which can lead to the activation of Nuclear factor-κB (NF-κB). Activated NF-κB rapidly transmits signals to the nucleus and acts as a transcription factor to enhance the transcription of tumour necrosis factor-α (*TNF-α*) and *IL-1β*. Subsequently, the secretion and release of IL-6, IL-8, as well as other proinflammatory cytokines and type I interferon-α (IFN-α) and interferon-β (IFN-β) were also increased, which can lead to the further amplification of inflammation [6–8]. These activities can ultimately lead to the activation of NF-κB and drive the release of proinflammatory cytokines and various inflammatory mediators, which activate lymphocytes and induce them to accelerate the synthesis of protein [9–13]. Almost, all TLRs can mediate downstream signal transduction via a MyD88-dependent pathway except for Toll-like receptor 3 (TLR3).

The porcine *MyD88* gene is located on pig chromosome 13, encodes 293 amino acids, and shares 87–88% homology with the human *MYD88* gene. It is widely expressed in various porcine tissues, especially in immune and intestinal tissues [14,15], and this expression pattern is related to its role in immunity and host defence. As a key adapter molecule in the TLR/IL-1R signalling pathway, MyD88 plays an important role in transmitting inflammatory signals, enhancing the intensity of inflammation and triggering the release of intestinal inflammatory mediators [16]. Considering the important role of MyD88 in protection against pathogen infection, we generated a *MyD88* RNAi silencing cell line by designing a shRNA expression vector, lentivirus transfection and effective screening. Therefore, we established a PK15 cell line with stable *MyD88* gene silencing. And we tested the effects of its down-regulated expression on the cytokine levels related genes and proinflammatory in TLR4 signalling pathway. This cell line will serve as a valuable reagent for further analyses of MyD88 functions and investigations of the mechanism of the TLR/IL-1R signalling pathway in porcine immune responses and host defence.

## MATERIALS AND METHODS

### Reagents and materials

The PK15 cell line was obtained from the A.T.C.C. fetal bovine serum (FBS), dulbecco's modified eagle medium (DMEM) and Opti-MEM medium were obtained from Gibco). The vectors pcDNA™6.2-GW/EmGFP-miR, pMD-18T, pcDNA3.1(+), pDONR221 and pLenti6.3/V5-DEST, and the vector construction kits BLOCK-iT™ Pol II miR RNAi Expression Vector Kit with EmGFP, Packaging Mix, T4 DNA ligase, high purity plasmid extraction kit, Lipofectamine 2000 and TRIzol were purchased from Invitrogen. Synergy Brands, Inc (SYBR) premix Ex*Taq* was purchased from Takara. Total protein extraction kit and BCA protein detection kit were purchased from Nanjing Keygen Technology. Primary antibodies–MyD88 (1:800), cluster of differentiation antigen 14 (CD14) (1:400), IFN-α (1:600), IL-1β (1:600), TLR4 (1:1000), TNF-α (1:1000) and β-actin (1:4000)–were purchased from Abcam. Second antibody (IgG-HRP, 1:3000) was purchased from Jackson. LPS was purchased from Sigma–Aldrich. Porcine IL-1β, TNF-α, IL-6, IL-8, IL-12, macrophage inflammatory protein (MIP)-1α and MIP-1β ELISA kits were purchased from AssayPro.

### Primer design and sequence synthesis

Based on *MyD88* gene coding sequence (NM_001099923.1), a total of four interference sequences (oligo) to target *MyD88* mRNA transcripts were designed (Table 1) and quantitative real-time PCR (qPCR) primers were designed based on the sequences of these genes from the pig available in the GenBank database using Primer Express 2.0 software (Table 2). All of primers were synthesized by Sangon Biotech.

**Table 1:** Single-strand DNA sequences for shRNAs designed for lentiviral vector construction In this table, italics indicate introduced enzyme loci: *Bam* HI recognizes the sequence of GATCC; *EcoR* I recognizes the sequence of AATTC; lowercase indicates the interference sequence and complementary sequence; and an underline represents the loop sequence.

Name	Oligo sequence (5′-3′)
1F	*TGCTG*ttcggcagtcctcttcaatgcGTTTTGGCCACTGACTGACgcattgaaggactgccgaa
1R	*CCTG*ttcggcagtccttcaatgcGTCAGTCAGTGGCCAAAACgcattgaagaggactgccgaaC
2F	*TGCTG*aagggatgctgctatctacagGTTTTGGCCACTGACTGACctgtagatcagcatccctt
2R	*CCTG*aagggatgctgatctacagGTCAGTCAGTGGCCAAAACctgtagatagcagcatcccttC
3F	*TGCTG*atctcggtcagacacacataaGTTTTGGCCACTGACTGACttatgtgtctgaccgagat
3R	*CCTG*atctcggtcagacacataaGTCAGTCAGTGGCCAAAACttatgtgtgtctgaccgagatC
4F	*TGCTG*taacagggatcagtcgtttctGTTTTGGCCACTGACTGACagaaacgagatccctgtta
4R	*CCTG*taacagggatctcgtttctGTCAGTCAGTGGCCAAAACagaaacgactgatccctgttaC
N-F	*TGCTG*aaatgtactgcgcgtggagacGTTTTGGCCACTGACTGACgtctccacgcagtacattt
N-R	*CCTG*aaatgtactgcgtggagacGTCAGTCAGTGGCCAAAACgtctccacgcgcagtacatttC

**Table 2:** List of primer pairs used for gene expression analysis by qPCR

Genes	Primer sequence 5′ → 3′: Forward/Reverse	Product length (bp)	Accession number
*TLR4*	CAGATAAGCGAGGCCGTCATT		
	TTGCAGCCCACAAAAAGCA	113	AB232527
*MyD88*	GTGCCGTCGGATGGTAGT		
	CAGTGATGAACCGCAGGAT	173	EU056736
*CD14*	CCTCAGACTCCGTAATGTG		
	CCGGGATTGTCAGATAGG	180	AB267810
*TNF-α*	CGACTCAGTGCCGAGATCAA		
	CCTGCCCAGATTCAGCAAAG	58	X54001
*IL-1β*	TGATTGTGGCAAAGGAGGA		
	TTGGGTCATCATCACAGACG	63	NM_001005149
*IFN-α*	CCTGGACCACAGAAGGGA		
	TCTCATGCACCAGAGCCA	92	X57191
*GAPDH*	ACATCATCCCTGCTTCTACTGG		
	CTCGGACGCCTGCTTCAC	188	AF017079
*Hsa-GAPDH*	GAAGGTCGGAGTCAACGGATT		
	CGCTCCTGGAAGATGGTGAT	228	NM_001289745

### Construction of interference vectors

Synthetic oligo DNA sequences were cloned into a pcDNA6.2-GW/EmGFP vector after annealing and ligation, products were transformed into DH5α competent cells, and multiple monoclonal plasmids were picked from solid culture plates that contained spectinomycin for sequencing. When the sequence of the product was confirmed to be correct, high-purity plasmid was extracted for subsequent use.

### Cell transfection and verification of interference efficiency

The interference and expression vectors were co-transfected into 293T cells to evaluate interference efficacy and the vectors with the best interference efficiency were selected. When the coverage of 293T cells cultured with DMEM (10% FBS) in six-well plates reached ∼80%, transfections were performed mediated by Lipofectamine 2000. Cell culture medium was replaced with fresh complete medium after it was warmed to 37°C in a 5% CO_2_ incubator for 6 h. Cells were then cultured overnight to observe the expression of GFP.

When cells had been transiently transfected for 48 h, total RNA from cells was extracted. And qPCR was used to detect the interference of target genes in cells according to the manufacturer's instructions. QPCR (PrimeScript™ RT Master Mix [Perfect Real Time], TAKARA) was carried out as follows: 10 μl SYBR Premix ExTaq™ II (2×), 0.4 μl PCR Forward Primer (10 μM), 0.4 μl PCR Reverse Primer (10 μM), 0.4 μl ROX Reference Dye II (50×), 2 μl cDNA, and 6.8 μL ddH_2_O was added to make the final volume 20 μL. The ABI 7500 system was used for the detection of qPCR. The relative levels of gene expression were calculated using the 2^−ΔΔ^*^C^*_T_ method, and the reference gene *GAPDH* was used to normalize gene expression levels [17]. And the interference efficiency was defined as 1−2^−ΔΔ^*^C^*_T_. Protein levels were normalized using the BCA kit (Nanjing Keygen Technology). The sample in each group was conducted by SDS/10% PAGE: 10 μl of protein-loading 160 V 70 min. Blotting: proteins were transferred to PVDF membranes and immunoblotted with antibodies to MyD88 (1:800), β-actin(1:4000) and IgG-HRP (1:3000).

### Lentiviral vector packaging and titre determination

Gateway recombination technology was used to select the vector with the optimal interference efficacy and it was cloned into a lentiviral vector by recombination. 293T cells were used for lentiviral packaging and virus titres were measured after concentrating the virus stock solution.

The restriction enzyme *Eag*I was used to linearize the interference plasmid by digestion, which was then purified. The interference sequence was cloned into pDONR 221 vectors using the BP recombination system, then was subcloned into a pLenti6.3/V5-DEST vector using a LR recombination system. Clones were picked after selection, and the recombinant plasmid clone was termed pLenti-*MyD88*-shRNA, validated by sequencing, and then plasmids were extracted from large bacterial cultures.

To produce bioactive shRNA virus, packaging mix (pLP1, pLP2 and pLP/VSVG) and pLenti-*MyD88*-shRNA plasmid were co-transfected mediated by Lipofectamine 2000. After incubation for 48 h, cell culture supernatants were collected and centrifuged at 1509 ***g*** for 10 min to pellet remaining cells and debris. Then, supernatants were filtered through 0.45 μm filters and the resultant virus stock solution was centrifuged at 50000 × ***g*** for 2 h. Finally, the supernatant was decanted and resuspended in Opti-MEM culture solution.

DMEM culture solution containing 8 μg/ml polybrene and 2% FBS was used to dilute the virus solution by 10-fold serial dilutions, and 100 μl culture solution of each 10-fold serial dilution was added to 96-well plates with 293T cells; serial dilutions were constructed in triplicate. After culturing for 24 h, the number of cells expressing GFP in each well was quantified using a fluorescence microscope. The virus titre (TU/ml) was calculated as follows: (average number of fluorescent cells per well)/(volume of virus solution).

### Infection of PK15 cells with virus

DMEM culture medium containing 10% FBS was used to culture PK15 cells until they reached the exponential growth phase in six-well plates, then the culture medium was replaced with fresh complete medium containing 8 μg/ml polybrene. Additionally, 20 μl of pLenti-*MyD88*-shRNA at a titre of 1×10^8^ TU/ml and a negative control virus solution were added to cell culture plates, and then cells expressing GFP were observed after culture for 24 h. And 10 μg/ml blasticidin was used and culture medium was replaced with fresh complete culture solution containing blasticidin every 24 h. After screening, positive cells were detected by fluorescence microscopy.

### Detection of the interference efficiency of MyD88 lentiviral vectors and measurements of the expression levels of TLR4 pathway-related genes

Continuous blasticidin screening till the expression level of lentivirus in PK15 cells was stabilized. And continuous subculture till the interference efficiency of *MyD88* gene in PK15 cells maintained stable level without significant change, PK15 cells were collected for total RNA extraction to detect the silencing efficiency of *MyD88* and measure the expression levels of TLR4 pathway-related genes. The method of detecting the transcript and translation level was the same with they mentioned in the previous section.

### LPS treatment and detection of downstream proinflammatory cytokines in cell culture supernatants

When three kinds of cells (Blank, NC and RNAi cells) were 80–90% confluent with six copies, three copies of them were exposed to 0.1 μg/ml LPS (Sigma–Aldrich) and culture supernatants were collected after inducing for 6 h (the concentration and treatment time of LPS were referred to the seminal work by Fitzgerald et al. [18]); the another three copies were cultured to more than 95% confluent and culture supernatants were collected. Then, ELISA was used to measure levels of seven proinflammatory cytokines (IL-1β, TNF-α, IL-6, IL-8, IL-12, MIP-1α and MIP-1β) in cell culture supernatants with ELISA kits purchased from AssayPro to analyse the effects of *MyD88* gene interference on proinflammatory cytokine levels. The assay procedure and standard curve generation were carried out according to the instructions provided with the kit.

### Statistical analysis

The relative levels of gene expression were calculated using the 2^−ΔΔ^*^C^*_T_ method, and the reference gene *GAPDH* was used to normalize gene expression levels [17]. Namely, Δ*C*_T_=the *C*_T_ values of target gene (*MyD88*)–the *C*_T_ values of housekeeping gene (*GAPDH*), and ΔΔ*C*_T_=the Δ*C*_T_ values of experimental group (the Δ*C*_T_ values of *MyD88* gene in each RNAi treatment respectively)–the Δ*C*_T_ values of reference group (the average of Δ*C*_T_ values of *MyD88* gene for BLANK group), then the relative expression level of MyD88 was defined as 2^−ΔΔ^*^C^*_T_. The expression level of MyD88 in BLANK group was defined as 1.0 (ΔΔ*C*_T_=0, 2^−ΔΔ^*^C^*_T_=1), and the interference efficiency was defined as 1−(2^−ΔΔ^*^C^*_T_). The method of one-way ANOVA provided with SPSS 16.0 software was used for comparative analyses of differential expression of genes and cytokines level differences between RNAi group and negative control group. Each independent experiment or treatment repeated for three times and the data were counted as *X*±S.D.

## RESULTS

### Vector construction and verification of interference efficacy

By verifying the sequencing of selected positive clones, we ultimately obtained four interference and one negative control plasmid vectors with the correct orientation, which we termed pcDNA6.2-GW/EmGFP-shRNA-1, pcDNA6.2-GW/EmGFP-shRNA-2, pcDNA6.2-GW/EmGFP-shRNA-3, pcDNA6.2-GW/EmGFP-shRNA-4 and pcDNA6.2-GW/EmGFP-shRNA-NC.

**Figure 1:**
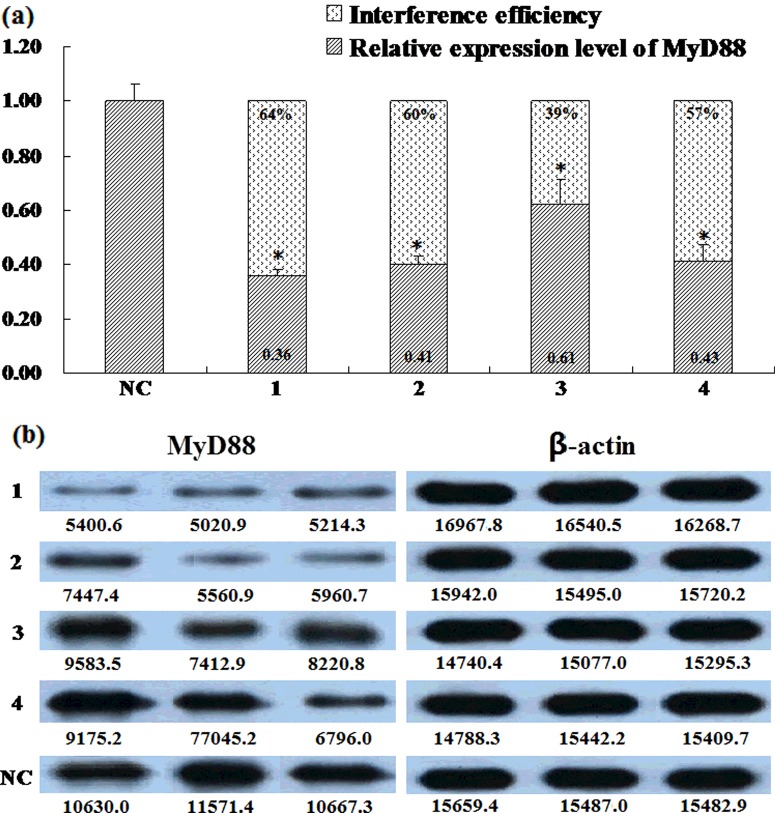
Detection of interference efficiency of *MyD88* gene The transcription levels (**a**) and the translation levels (**b**) of *MyD88* gene. Measurements of *MyD88* mRNA transcripts and interference efficiency were detected by qPCR and the translation levels of *MyD88* were detected by Western blot when cells had been transfected for 48 h and total RNA and protein were extracted; four different interference vectors (1, 2, 3 and 4) were tested; NC, negative control vector. The expression level of *MyD88* in NC group was defined as 1.0, and data were derived from three independent experiments. The mean values ± S.D. for three repeats of each group are shown in results of transcription levels. Significant difference between the interference group and the negative control group was tested by the method of one–way ANOVA; *P<0.05. And the interference efficiency was defined as 1- expression level.

The interference and overexpression vectors were used to co-transfect 293T cells, and qPCR as well as Western blot were used to measure the transcript and translation levels of *MyD88*. We found that the interference efficacy of pcDNA6.2-GW/EmGFP-shRNA-1 was the highest whose relative expression level of porcine *MyD88* of 0.36 with an interference efficacy of 0.64 and the Western blot results confirmed the interference effect (Figure 1), so we used this vector for lentivirus packaging.

**Figure 2:**
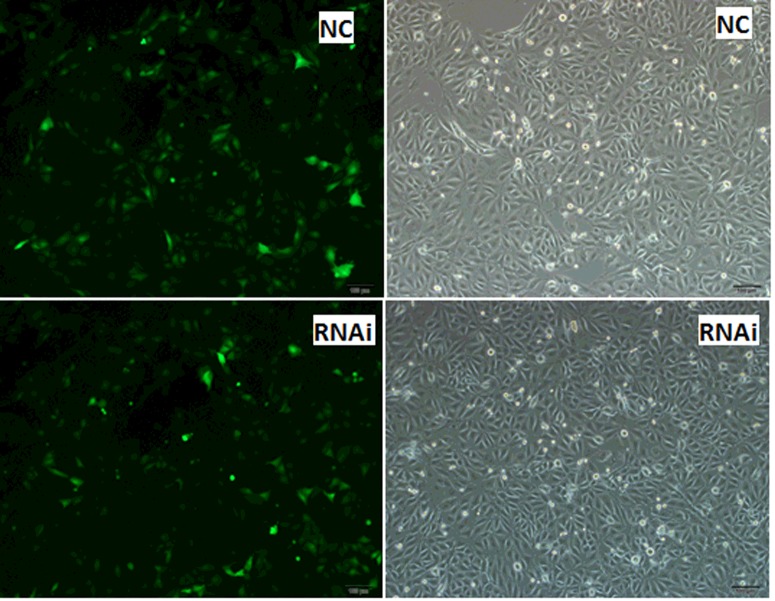
The preliminary integration of lentivirus in PK15 cells The expression of GFP in PK15 cells infected by lentivirus for 24 h without blasticidin screening. The expression rate number of fluorescent protein of RNAi was 92.38%, and NC was 90.25%. The expression rate of GFP (%) was calculated as follows: (average number of fluorescent cells in three different fields of fluorescence microscope of three treatments repeats)/(average number of total number of three treatments repeats). The RNAi method involved infection of cells by lentivirus virus solution; NC, cells infected by negative control virus. Cells were observed under an inverted fluorescence microscope; magnification, 100×.

**Figure 3:**
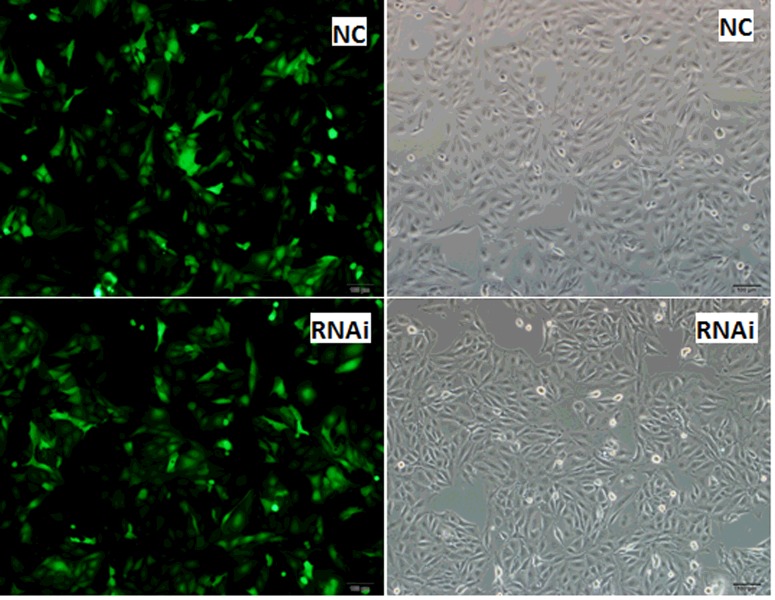
The stable expression of GFP in PK15 cells after continuous screening The expression of GFP in PK15 cells screened by blasticidin for 72 h. Namely, PK15 cells were infected by lentivirus for 98 h, which aimed to illustrate the expression stability of lentivirus. The expression rate number of fluorescent protein of RNAi was 92.38%, and NC was 90.25%. The expression rate of GFP (%) was calculated as follows: (average number of fluorescent cells in three different fields of fluorescence microscope of three treatments repeats)/(average number of total number of three treatments repeats). The RNAi method involved infection of cells by lentivirus virus solution; NC, cells infected by negative control virus. Cells were observed under an inverted fluorescence microscope; magnification, 100×.

The expression of GFP was observed under an inverted fluorescence microscope after PK15 cells were infected with pLenti-*MyD88*-shRNA and negative control virus for 24 h (Figure 2). After blasticidin screening for 72 h, the expression level of lentivirus in PK15 cells was stabilized (Figure 3). We found that pLenti-*MyD88*-shRNA and the negative control virus could infect the PK15 cell line with the higher expression efficiency compared with cells without transfection, and the proportion of positive cells increased after blasticidin screening. These findings indicated that the interference vector could achieve a better transduction, so we used it for subsequent assays.

### Interference efficacy of MyD88 lentiviral vectors and the expression levels of TLR4 pathway-related genes

**Figure 4:**
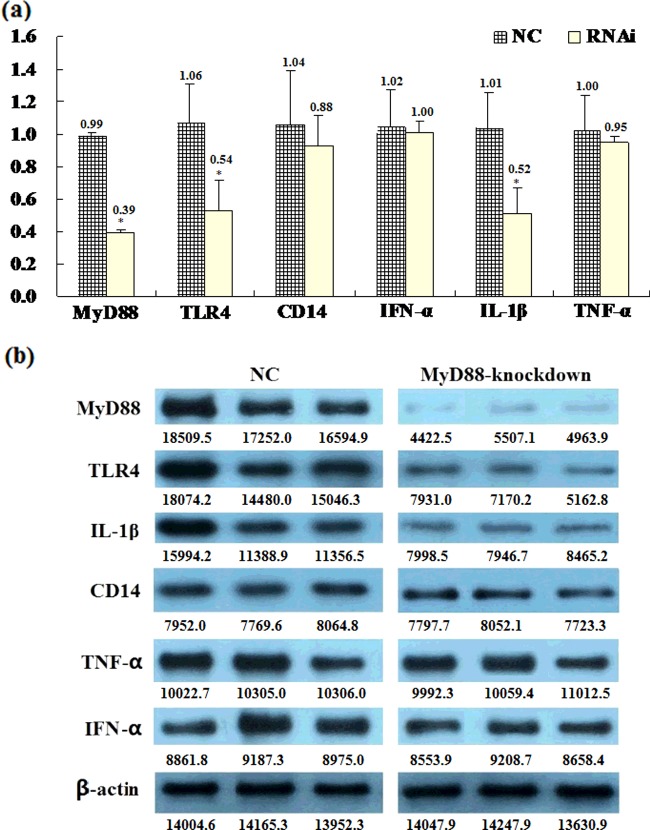
The effect of *MyD88* silencing on the expression of TLR4 signaling pathway-related genes The transcription levels (**a**) and the translation levels (**b**) of genes in TLR4 signalling pathway in PK15 cells with stable *MyD88* silencing when screened by blasticidin for 72 h; NC, negative control group; RNAi, *MyD88* interference group. The transcription levels of these genes were analysed by qPCR. The expression level of *MyD88* in negative control group was defined as 1.0, and data were derived from three independent experiments±S.D. for groups of three replicates per each group. The mean values were used to analyse significant difference between the interference group and the negative control group by the method of one-way ANOVA, *P<0.05. The translation levels were analysed by Western blot and β-actin was defined as internal reference. Each group has three samples loaded from individual cells.

After blasticidin screening for 72 h, the expression level of lentivirus in PK15 cells was stabilized. With continuous subculture, the interference efficiency of *MyD88* gene in PK15 cells maintained ∼ 61% without distinct change (Figure 4a), which indicated that we have successfully established a PK15 cell line with stable *MyD88* gene silencing. Then, qPCR was used to detect the transcription and translation levels of TLR4 pathway-related genes (Figure 4). The mRNA expression level of *MyD88* in negative control group was defined as 1.0, and data were derived from three independent experiments±S.D. for groups of three replicates per group. We found that the mRNA transcript levels of *TLR4* and *IL-1β* in the interference group were significantly reduced, whereas there was no significant change in the mRNA transcript levels of *CD14*, *IFN-α* or *TNF-α* (Figure 4a). And the protein expression level of MyD88, TLR4 and IL-1β was significantly induced after *MyD88* gene silencing whereas there was no significant change of the expression in *CD14*, *IFN-α* or *TNF-α*, which verified it's consistent with corresponding mRNA expression level (Figure 4b).

### Detection of proinflammatory cytokines in cell culture supernatants

The detection of proinflammatory cytokines in cell culture supernatants was conducted strictly according to the instructions of ELISA kits. A standard curve for each proinflammatory cytokine was derived from a quadratic regression equation according to specification, and the concentration of cytokines in supernatants with different treatments was figured by standard curve equation (Supplementary Figure S1). Data were analysed by SPSS 16 and shown as *X*±S.D. And the general linear model (GLM) was used for comparative analyses of cytokines level differences under different treatments. We found that when cells were not induced by LPS, the levels of IL-8 were significantly up-regulated in cell culture supernatants with *MyD88* silencing whereas there were no significant changes in the concentrations of the other six proinflammatory cytokines (Table 3). However, the global level of seven proinflammatory cytokines up-regulated when cells were induced by LPS for 6 h. As Table 4 showed, the level of IL-1β, TNF-α, IL-6, IL-8 and IL-12 of Blank and NC group up-regulated more significantly than RNAi group (*P*<0.05).

**Table 3:** Release levels of downstream proinflammatory cytokines in cell culture supernatants without LPS stimulation Different superscript letters within the same row indicate means that differ significantly (*P*<0.05); means with the same superscript letter within the same row or without a superscript letter do not differ significantly (*P*>0.05).

Cytokines (ng/l)	Blank control group	Negative control group	RNAi group
IL-1β	271.55±7.56	277.21±6.35	243.77±6.38
TNF-α	62.39±0.28	59.44±0.16	64.93±0.54
IL-6	13.67±1.71	13.76±2.17	15.71±0.74
IL-8	34.31±2.41a	34.03±2.14a	48.54±7.49^b^
IL-12	7.75±0.03	7.77±0.03	7.76±0.03
MIP-1α	65.09±0.34	65.18±0.42	65.89±0.71
MIP-1β	111.53±3.48	111.72±4.35	110.61±1.34

**Table 4:** Release levels of downstream proinflammatory cytokines in cell culture supernatants when cells were induced by LPS for 6 h Different superscript letters within the same row indicate means that differ significantly (*P*<0.05); means with the same superscript letter within the same row or without a superscript letter do not differ significantly (*P*>0.05).

Cytokines (ng/l)	Blank control group	Negative control group	RNAi group
IL-1β	350.37±10.49a	337.25±8.96a	250.38±6.57^b^
TNF-α	100.42±5.36a	99.18±3.34a	70.38±3.25^b^
IL-6	20.43±2.28a	20.53±2.58a	15.79±2.16^b^
IL-8	70.31±4.35a	69.31±5.81a	48.58±4.12^b^
IL-12	12.23±1.58a	11.99±2.01a	7.79±0.04^b^
MIP-1α	70.66±3.42	71.12±4.35	67.07±0.81
MIP-1β	130.27±5.19	127.57±4.33	112.34±3.86

## DISCUSSION

Studies have shown that *MyD88* knockout mice exhibit resistance to doses of LPS that would kill most wild-type mice [19]. In *Drosophila*, overexpression of *MyD88* can induce the production of antimicrobial peptides, whereas antimicrobial peptide levels are reduced in *MyD88* mutants [20]. Studies also have shown that *MyD88* plays a significant regulatory role in the process of signal transducer and activator of transcription 3 (STAT3) phosphorylation and LPS-induced suppressor of cytokine signaling 3 (SOCS3) expression, and it was found that *MyD88* knockout mice were resistant to LPS-induced appetite suppression [21]. Additionally, studies of neurobiology have shown that *MyD88* defects can cause cognitive and behavioural disorders in mice [22]. Loiarro et al. [23] successfully blocked the dimerization of *MyD88* by synthesizing a peptide mimetic of the TIR domain of MyD88. Advances in molecular biology have led to the development of many gene modification techniques that can be used for gene function analyses. Among them, the lentiviral vector method has been frequently used to generate transgenic cells, embryos and live animals. Studies of *MyD88* gene function have largely utilized gene knockout technology to produce *MyD88^−/−^* animals, allowing the role of MyD88 in signalling pathways to be studied. After the *MyD88* gene is deleted, the signalling pathways dependent on *MyD88* are interrupted and immune signal transduction is altered. Although the vital function of a target gene can be highlighted by this approach, changes in the immune response caused by differences that alter the expression levels of *MyD88* cannot be analysed. However, RNAi can partially reduce the expression of a target gene, thereby providing the possibility of functional analyses of genes with different expression levels. Additionally, RNAi technology can also be used to study virus defence, disease treatments and other research subjects [24]. Considering the important role of MyD88 in the TLR/IL-1R signalling pathway, the present study successfully constructed and developed a lentivirus-mediated *MyD88* gene interference vector using RNAi, which showed good infection and interference effects. We obtained PK15 cell lines for which the silencing efficiency of *MyD88* mRNA transcripts reached 61%, thereby establishing it as an ideal reagent for functional studies of the *MyD88* gene. These findings also provide a basis for the functional study of TLR4 signalling pathway-related genes during an immune response.

The present study also showed that *TLR4* and *IL-1β* exhibited down-regulated levels of expression after *MyD88* gene expression was knocked down, whereas there was no significant change in the expression of *CD14*, *IFN-α* and *TNF-α* mRNA transcripts. And the results of protein expression verified the accuracy of the mRNA expression level. Changes in levels of *MyD88* expression can affect both the expression of pathways downstream of *IL-1β*, but also affect the expression of genes upstream of *TLR4*, indicating that *MyD88* is a key adaptor molecule in TLR/IL-1R signalling pathways. It may also contribute to immune regulation through interactions with key genes involved in the immune response pathway that are activated by stimulation with various pathogens. There were no significant changes in the expression levels of *CD14*, *IFN-α* or *TNF-α*, indicating that the down-regulation of *MyD88* did not cause widespread changes in all of the genes involved in these pathways, and these differences in altered expression levels may be related to *MyD88*-dependent compared with *MyD88*-independent pathways involved in the TLR/IL-1R signalling pathway. TRIF (TIR-domain-containing adaptor inducing interferon-β), TRAM (TRIF-related adaptor molecule) and TIRAP/Mal (TIR-domain-containing adaptor protein, *MyD88*-adaptor-like) also play important roles in *MyD88*-independent signalling pathways and utilize a TIR domain [25,26].

ELISA findings showed that among seven different proinflammatory cytokines, only levels of IL-8 significantly increased, whereas there were no significant changes in the levels of the other cytokines when cells were not induced by LPS. However, the level of IL-1β, TNF-α, IL-6, IL-8 and IL-12 of Blank and NC group up-regulated more significantly than RNAi group when cells were induced by LPS for 6 h. These results revealed that the immune signalling pathways were in a relatively silent state under normal physiological conditions, for which the levels of proinflammatory cytokines were low and relatively stable. When cells were stimulated by LPS, the immune signalling pathways were activated and triggered up-regulation of downstream proinflammatory cytokines, which finally caused inflammatory response. IL-1β, TNF-α, IL-6, IL-8 and IL-12 are important proinflammatory cytokines that can be released after NF-κB activation by the TLR4 signalling pathway, the down-regulation of these cytokines illustrated that the *MyD88* silencing could reduce the TLR4 signal transduction that inhibited the release of proinflammatory cytokines and finally leaded to immunosuppression [27]. IL-8 belongs to the C-X-C subfamily and can simultaneously act as a proinflammatory cytokine and chemokine to activate neutrophils, chemotactic T cells and basophils and also plays an important regulatory role in inflammatory and immune processes [28–30]. Compared the level of IL-8 under the different treatments with LPS induce or not, we speculated that the significant up-regulation of IL-8 without LPS induce in cell culture supernatants was not caused by *MyD88-*dependent signalling pathway because the level of RNAi group up-regulated less significantly than Blank and NC group when cells were induced by LPS, which suggested that the interference of *MyD88* gene could inhibit the release of IL-8. Further detection and analysis can be conducted for the mechanism of *MyD88-*independent signalling pathway up-regulating the level of IL-8.

## Abbreviations

CD14: cluster of differentiation antigen 14
DMEM: dulbecco's modified eagle medium
FBS: fetal bovine serum
IFN-α: interferon-α
IFN-β: interferon-β
IL: Interleukin
IRAK: Interleukin receptor associated kinase
LPS: lipopolysaccharide
MIP: macrophage inflammatory protein
MyD88: myeloid differentiation protein 88
NC: Negative control
NF-κB: Nuclear factor-κB
qPCR: quantitative real-time PCR
SOCS3: Suppressor of cytokine signaling 3
STAT3: Signal transducer and activator of transcription 3
SYBR: Synergy Brands, Inc
TIR: Toll/Interleukin-1 receptor
TLR: Toll-like receptor
TLR4: Toll-like receptor 4
TNF-α: tumour necrosis factor-α
TRAF-6: TNF receptor associated factor-6
